# Regional Contrasts of the Warming Rate over Land Significantly Depend on the Calculation Methods of Mean Air Temperature

**DOI:** 10.1038/srep12324

**Published:** 2015-07-22

**Authors:** Kaicun Wang, Chunlüe Zhou

**Affiliations:** 1 College of Global Change and Earth System Science, Beijing Normal University, Beijing, 100875, China; 2 Joint Center for Global Change Studies, Beijing 100875, China

## Abstract

Global analyses of surface mean air temperature (*T*_*m*_) are key datasets for climate change studies and provide fundamental evidences for global warming. However, the causes of regional contrasts in the warming rate revealed by such datasets, i.e., enhanced warming rates over the northern high latitudes and the “warming hole” over the central U.S., are still under debate. Here we show these regional contrasts depend on the calculation methods of *T*_*m*_. Existing global analyses calculate *T*_*m*_ from daily minimum and maximum temperatures (*T*_*2*_). We found that *T*_*2*_ has a significant standard deviation error of 0.23 °C/decade in depicting the regional warming rate from 2000 to 2013 but can be reduced by two-thirds using *T*_*m*_ calculated from observations at four specific times (*T*_*4*_), which samples diurnal cycle of land surface air temperature more often. From 1973 to 1997, compared with *T*_*4*_, *T*_*2*_ significantly underestimated the warming rate over the central U.S. and overestimated the warming rate over the northern high latitudes. The ratio of the warming rate over China to that over the U.S. reduces from 2.3 by *T*_*2*_ to 1.4 by *T*_*4*_. This study shows that the studies of regional warming can be substantially improved by *T*_*4*_ instead of *T*_*2*_.

Near surface air temperature over land has a significant diurnal cycle, primarily determined by diurnal variation of surface net radiation, i.e., the sum of the net solar (incident minus reflected) and the net longwave (absorbed downwelling minus emitted upwelling) radiation at the surface. During the nighttime, surface net radiation is negative, and near surface air temperature decreases with time, reaching its minimum (*T*_*min*_) in the early morning. Surface net radiation becomes positive during the daytime due to the absorption of incident solar radiation at the surface, and this surface net radiation is partitioned into latent heat and sensible heat fluxes. As the air above the surface is directly heated by the sensible heat flux, air temperature reaches its maximum (*T*_*max*_) in the early afternoon^1^.

*T*_*max*_ and *T*_*min*_ have been operationally observed at weather stations globally since the middle of the 19th century^2^,^3^. Their mathematical average *T*_*2*_ = (*T*_*max*_ + *T*_*min*_)/2 has been taken as the standard method for calculating mean air temperature (*T*_*m*_)^4^ and has been the backbone of current global analyses of air temperature over land^5^,^6^,^7^. It is well known that *T*_*2*_ is different from the true *T*_*m*_, i.e., mean air temperature calculated from 24 hourly air temperature observations (*T*_*24*_)^8^,^9^.

It has been assumed that *T*_*2*_ can be used to accurately depict annual anomalies and trends in *T*_*m*_^10^,^11^. However, recent studies have shown that *T*_*2*_ trends can be significantly biased. It was found that *T*_*2*_ trends have significant biases of 25% (one standard deviation) at a grid scale size of 5°×5°^12^. This is because *T*_*2*_ only samples the diurnal cycle of air temperature twice daily, and *T*_*2*_−*T*_*24*_ changes with the land surface conditions (e.g., wetness and vegetation coverage). Surface conditions dominate the partitioning of surface net radiation into the latent heat and sensible heat fluxes. Sensible heat fluxes directly heat air above the surface and largely determine the diurnal curve of air temperature above land^13^.

Air temperature has been measured more frequently at weather stations since the 1950 s, with the establishment of the World Meteorological Organization (WMO). Under the guidelines of the WMO, air temperature at 00:00, 06:00, 12:00 and 18:00 UTC (Coordinated Universal Time) has been measured operationally at weather stations. *T*_*m*_ can also be calculated from these four observations (*T*_*4*_). We have accumulated more than 60 years of *T*_*4*_ data at global weather stations. Since the 1990 s, hourly air temperature has been widely available at global weather stations with the development of automatic weather stations^3^. However, to remain homogenous, the century-long global analyses of *T*_*m*_ still rely on *T*_*2*_^4^,^11^.

In this study, we first evaluate and compare the uncertainty of *T*_*2*_ and *T*_*4*_ in depicting the trend in *T*_*m*_ from 2000 to 2013. We use *T*_*24*_ as the true *T*_*m*_ to serve as a comparison^8^,^9^. We calculate *T*_*2*_, *T*_*4*_ and *T*_*24*_ from hourly observations from the Integrated Surface Hourly Database (ISD)^14^ and the Global Historical Climatology Network (GHCN) daily database^15^ released by the National Climate Data Center (NCDC). In evaluating the trend bias of *T*_*2*_ (or *T*_*4*_), we select the exact same stations and time periods of *T*_*2*_ (or *T*_*4*_) and *T*_*24*_. We found that there are more than 3000 globally distributed stations from which *T*_*2*_, *T*_*4*_, and *T*_*24*_ were available for more than 84 months during 2000 to 2013.

## Results

**Figure 1:**
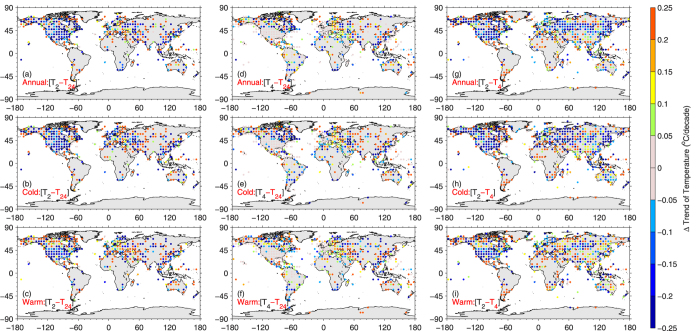
Trend biases in mean air temperatures (*T*_*m*_) calculated from different methods: *T*_*2*_, calculated from daily maximum and minimum temperature (*T*_*2*_, left column), and *T*_*4*_, calculated from four observations at 00:00, 06:00, 12:00, and 18:00 UTC (middle column). *T*_*m*_s calculated from 24 hourly air temperatures (*T*_*24*_) were used as reference data. Trend differences between *T*_*2*_ and *T*_*4*_ were also shown in the right column. The cold season is defined as November to April over the Northern Hemisphere and May to October over the Southern Hemisphere, and the warm season is defined as May to October over the Northern Hemisphere and November to April over the Southern Hemisphere. *T*_*2*_ and *T*_*4*_ at 6000 stations and *T*_*24*_ at 3000 stations from 2000 to 2013 from the Global Historical Climatology Network (GHCN) daily database and the Integrated hourly Surface Data (ISD) were used here. *T*_*2*_ and *T*_*24*_ at identical time periods at a station were selected to calculate monthly anomalies. Monthly anomalies at the stations were aggregated into a 1°×1° grid and then into 5°×5° grid monthly anomalies, from which trends were calculated and shown in the left column. This is the same for the middle and right columns. The Figures were produced by MATLAB software.

**Figure 2:**
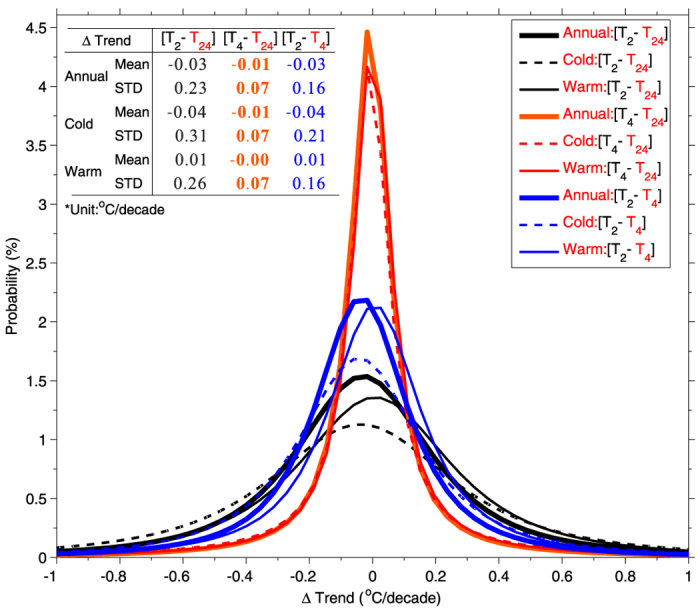
Fitted histograms of annual, cold season and warm season trend differences: *T*_*2*_−*T*_*24*_ (black), *T*_*4*_−*T*_*24*_ (red), and *T*_*2*_−*T*_*4*_ (blue) at 5°×5° grid as shown in Fig. 1. Statistical parameters of the trend differences, including mean and standard deviation (STD), are shown here. The data used here are the same as in Fig. 1. From 2000 to 2013, the global averaged trend in the mean air temperature over land is slightly underestimated by 0.03 °C/decade by *T*_*2*_. At a 5°×5° grid scale, the trend in *T*_*2*_ has a significant standard deviation error of 0.23 °C/decade. This error can be substantially reduced by more than two-thirds by using *T*_*4*_.

Figures 1 and 2 show that *T*_*2*_ demonstrates significant uncertainty in quantifying the trend in *T*_*m*_. For the global average, *T*_*2*_ underestimated the trend in global mean *T*_*m*_ by 0.03 °C/decade compared with *T*_*24*_ from 2000 to 2013. This indicates that the recent hiatus of warming was slightly overestimated by using current global analyses of *T*_*2*_. This mean bias of *T*_*2*_ can be reduced to 0.01 °C/decade by using *T*_*4*_. This also partly explains the discrepancy between the observed and modelled warming hiatus during the most recent 15 years^16^. Current climate models run at a time step of approximately 30 minutes and calculate *T*_*m*_ from these calculations, which is nearly equal to *T*_*24*_, whereas reference data provided by existing global analyses use *T*_*2*_ over land.

At a 5°×5° grid scale, *T*_*2*_ has a standard deviation error of 0.23 °C/decade in quantifying the trend in *T*_*m*_ (Fig. 2). This error is 0.31 °C/decade during cold seasons and is 0.26 °C/decade for warm seasons. This is because the diurnal cycle of air temperature changes with the surface conditions^12^. The error is higher during cold seasons because the impact of land-atmosphere interactions on diurnal variation in the surface air temperature is stronger during cold seasons. The surface is drier with lower vegetation coverage during cold seasons, and therefore, a higher fraction of surface net radiation is partitioned into the sensible heat flux, which directly heats the air above the surface.

This standard deviation error of the trend in *T*_*2*_ can be substantially reduced by two-thirds using *T*_*4*_ (Fig. 2). The primary reason for this is that *T*_*4*_ samples the diurnal cycle of air temperatures four times a day, whereas *T*_*2*_ only samples twice. Figures 1 and 2 indicate that *T*_*4*_ is more suitable to study the trend in *T*_*m*_ than *T*_*2*_.

We therefore compare the trends in *T*_*m*_ over land on a global scale from 1973 to 1997, the enhanced warming period. We select 1973 as the start year of the period because the *T*_*4*_ data released by ISD have much better spatial coverage since 1973. On average, compared with *T*_*4*_, *T*_*2*_ overestimated the global mean warming rate by 0.02 °C/decade from 1973 to 1997, the enhanced warming period (Table 1).

**Figure 3:**
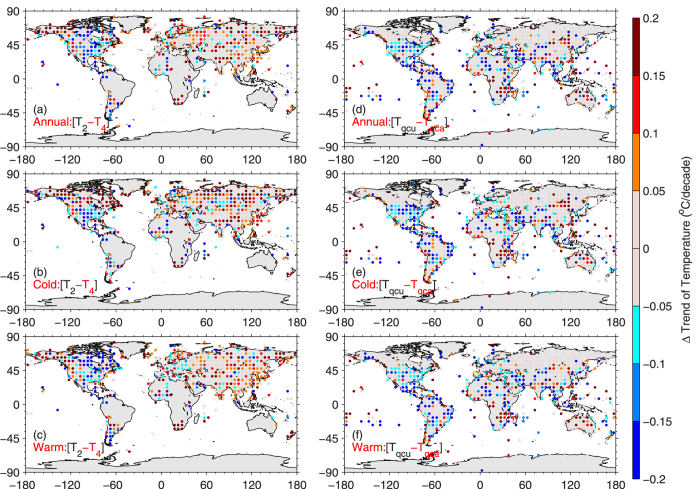
Differences in mean air temperature trends calculated using different methods (left column): *T*_*2*_ was calculated from daily maximum and minimum temperature, and *T*_*4*_ was calculated from four observations at 00:00, 06:00, 12:00, and 18:00 UTC. Definitions of the cold season and warm season can be found in the caption of Fig. 1. Hourly observations of air temperatures from 1973 to 1997 at 3300 stations were used here. *T*_*2*_ and *T*_*4*_ at identical time periods at a station were selected to calculate monthly anomalies. Monthly anomalies at the stations were aggregated into a 1°×1° grid and then into 5°×5° grid monthly anomalies, from which trends were calculated and shown. Compared with *T*_*4*_, *T*_*2*_ significantly underestimated the warming rate over the central U.S. and overestimated warming over the northern high latitudes and mainland China. For comparison, the differences in trends calculated from monthly raw *T*_*2*_ (*T*_*qcu*_) and monthly homogenized adjusted *T*_*2*_ (*T*_*qca*_) are shown in the right column. The Figures were produced by MATLAB software.

Compared with *T*_*4*_, *T*_*2*_ overestimates the warming rate over the northern high latitudes, which is more obvious in cold seasons. In particular, the trend of *T*_*2*_ is 0.06 °C/decade (27%) higher than that of *T*_*4*_ over Northern Siberia (Table 1). The overestimation is stronger during cold seasons. The enhanced warming rate over these regions has been identified in the IPCC reports^17^,^18^. Different mechanisms have been proposed to explain the enhanced warming rate over these regions^19^,^20^. It still cannot be fully explained, and the state-of-the-art global climate models have not been able to fully reproduce the enhanced warming rate^21^. Figure 3 and Table 1 show that the use of *T*_*2*_ at least partially explains the reported enhanced cold season warming over the northern high latitudes.

The regional warming rate over land significantly depends on the calculation methods of *T*_*m*_. Compared with *T*_*4*_, *T*_*2*_ also substantially overestimates the warming rate by 0.06 °C/decade (25%) over China from 1973 to 1997. As a result, the ratio of the warming rate in China to that in the U.S. is 2.3 for *T*_*2*_ and 1.4 for *T*_*4*_. In the central U.S., *T*_*2*_ has a negative trend of −0.05 °C/decade, whereas *T*_*4*_ is 0.03 °C/decade. This partially explains the reported “warming hole” over the central U.S. during this period^22^. It is known that the variation in recording time has introduced a spurious negative trend in *T*_*max*_, *T*_*min*_, and *T*_*2*_^23^. A homogenization method has been developed to homogenize monthly *T*_*2*_^24^,^25^. Figure 3 shows that the homogenization has a negligible impact on the *T*_*2*_ trend over the northern high latitudes. The homogenization only partly corrects the underestimation of *T*_*m*_ trend in *T*_*2*_ over the U.S.

## Discussion

This study shows that, compared with *T*_*4*_, current widely used datasets based on *T*_*2*_ overestimate the global mean warming rate by 0.02 °C/decade during the enhanced warming period (1973–1997) and underestimate the warming rate by 0.03 °C/decade during the hiatus period of warming (2000–2013). *T*_*2*_ has difficulty in accurately reflecting the impact of land-atmosphere interactions on *T*_*m*_^26^,^27^. The use of *T*_*2*_ also partly explains the reported enhanced cold season warming rates in the northern high latitudes and the “warming hole” over the central U.S. The regional contrast in the warming rate between the U.S. and China is therefore substantially decreased if *T*_*4*_ is used rather than *T*_*2*_. The bias of *T*_*2*_ also partly explains the discrepancy between the observed and modelled recent warming hiatus.

Because we have accumulated more than 60 years of *T*_*4*_ data, it is essential to use *T*_*4*_ to conduct a global analysis of *T*_*m*_, which will substantially improve upon previous studies of regional climate change detection and attribution. This can be performed by calculating *T*_*m*_ using the recently available Integrated Surface Hourly Database^14^ and the GHCN daily database^15^, which are available at ftp://ftp.ncdc.noaa.gov/pub/data/. Both projects have collected hourly or daily data at tens of thousands of weather stations. However, these data sources have not been fully used in current global *T*_*m*_ analyses^5^,^6^,^7^,^11^. The new analyses of global mean *T*_*m*_ will substantially improve our understanding of spatial warming patterns and regional climate change.

## Data and Method

This paper investigates *T*_*m*_ calculated by different methods. Mean air temperature (*T*_*2*_) calculated from daily maximum (*T*_*max*_) and minimum (*T*_*min*_) air temperatures has been widely accepted. To calculate *T*_*2*_, we use *T*_*max*_ and *T*_*min*_ from the Global Historical Climatology Network (GHCN) daily database^15^, which has the best spatial and temporal coverage. Air temperature observations at specific times, i.e., 00:00, 06:00, 12:00, and 18:00, from the Integrated Surface Hourly Database (ISD)^14^ are used to calculate *T*_*4*_. Hourly air temperatures from ISD are used to calculate *T*_*24*_, which is regarded as the true *T*_*m*_^8^,^9^, and to evaluate the uncertainty of *T*_*2*_ and *T*_*4*_.

Raw data of *T*_*max*_ and *T*_*min*_ have been known to be impaired by changes in recording time^3^. This is particularly important for the U.S.^23^. To address this issue, the pairwise comparison method has been used to homogenize the monthly *T*_*m*_^25^, which has been demonstrated to perform well over the U.S.^24^. However, homogenized and adjusted *T*_*2*_ (*Tqca*) are available at fewer stations than the daily base^5^,^15^. To make comparisons between *T*_*2*_, *T*_*4*_, and *T*_*24*_ at exactly the same time and stations, we calculate these *T*_*m*_s from raw hourly and daily data and then calculate their monthly values. In Fig. 3, we show the impact of homogenization is much less than that in different calculation methods.

Because *T*_*24*_ has only been widely available since 2000, we first evaluate the uncertainty of *T*_*2*_ and *T*_*4*_ in depicting *T*_*m*_ trend by comparison with *T*_*24*_ from 2000 to 2013. Our results show that *T*_*4*_ can substantially reduce the uncertainty of *T*_*2*_ in quantifying the *T*_*m*_ trend. We then compare the trends in *T*_*2*_ and *T*_*4*_ from 1973 to 1997 (the enhanced warming period), which shows that spatial contrasts in the warming rate over land significantly depends on the definitions of *T*_*m*_, i.e., *T*_*2*_ vs. *T*_*4*_.

## Additional Information

**How to cite this article**: Wang, K. C. and Zhou, C. Regional Contrasts of the Warming Rate over Land Significantly Depend on the Calculation Methods of Mean Air Temperature. *Sci. Rep.* **5**, 12324; doi: 10.1038/srep12324 (2015).

## Figures and Tables

**Table 1:** This table summarizes the linear trends from 1973 to 1997 of mean air temperature (*T*_*m*_) calculated from daily maximum and daily minimum temperature (*T*_*2*_) and from four observations of temperature at 00:00, 06:00, 12:00, 18:00 UTC time (>*T*_*4*_).

	**Globe**		**North Hemisphere**		**South Hemisphere**		**North America**		**Europe**		**USA**		**China**		**Alaska**		**Central USA**		**North Siberia**	
	***T***_***2***_	***T***_***4***_	***T***_***2***_	***T***_***4***_	***T***_***2***_	***T***_***4***_	***T***_***2***_	***T***_***4***_	***T***_***2***_	***T***_***4***_	***T***_***2***_	***T***_***4***_	***T***_***2***_	***T***_***4***_	***T***_***2***_	***T***_***4***_	***T***_***2***_	***T***_***4***_	***T***_***2***_	***T***_***4***_
Annual	**0.14**	**0.13**	**0.16**	**0.14**	**0.06**	**0.06**	0.09	0.11	0.18	0.17	0.09	0.12	**0.21**	**0.17**	**0.20**	**0.18**	−0.05	0.03	**0.17**	0.15
Cold	**0.16**	**0.14**	**0.18**	**0.15**	**0.05**	**0.05**	0.10	0.09	0.14	0.14	0.16	0.21	**0.31**	**0.25**	0.17	0.19	0.01	0.10	**0.28**	0.22
Warm	**0.13**	**0.12**	**0.14**	**0.13**	**0.03**	**0.04**	0.06	0.09	**0.21**	**0.21**	0.04	0.06	**0.15**	0.11	0.14	0.13	−0.06	0.01	**0.14**	0.12

The trends in this table have the same unit of °C/decade. Three regional averages of Alaska ([180°W:130°W, 55°N:75°N]), central USA ([105°W:75°W, 30°N:50°N]), and North Siberia ([60°E:180°E,60°N:80°N]) were provided in this table. The numbers in bold black indicate the linear trend passes the α = 0.05 level using a student’s t confidence test. Compared with *T*_*4*_, *T*_*2*_ overestimated the warming trend in China and underestimated the warming rate in USA. The ratio of the warming rate in China to that in USA is 2.3 if *T*_*2*_ was used to calculate the warming rates and 1.4 if *T*_*4*_ is used instead. The difference between the trends in *T*_*2*_ and T_4_ partly accounts for the reported “warming hole” in the central USA.
